# Effect of Methamphetamine on the Microglial Damage: Role of Potassium Channel Kv1.3

**DOI:** 10.1371/journal.pone.0088642

**Published:** 2014-02-12

**Authors:** Jun Wang, Wenyi Qian, Jingli Liu, Jingjing Zhao, Pan Yu, Lei Jiang, Jing Zhou, Rong Gao, Hang Xiao

**Affiliations:** 1 Key Lab of Modern Toxicology, Ministry of Education. Department of Toxicology, School of Public Health, Nanjing Medical University, Nanjing, China; 2 Department of Emergency Medicine, the First Affiliated Hospital of Nanjing Medical University, Nanjing, China; 3 Departments of Experimental Medicine, Nanjing Drum Tower Hospital, Nanjing Medical University, Nanjing, China; Virginia Commonwealth University, United States of America

## Abstract

Methamphetamine (Meth) abusing represents a major public health problem worldwide. Meth has long been known to induce neurotoxicity. However, the mechanism is still remained poorly understood. Growing evidences indicated that the voltage-gated potassium channels (Kv) were participated in neuronal damage and microglia function. With the whole cell patch clamp, we found that Meth significantly increased the outward K^+^ currents, therefore, we explored whether Kv1.3, one of the major K^+^ channels expressed in microglia, was involved in Meth-induced microglia damage. Our study showed that Meth significantly increased the cell viability in a dose dependent manner, while the Kv blocker, tetraethylamine (TEA), 4-Aminopyridine (4-AP) and Kv1.3 specific antagonist margatoxin (MgTx), prevented against the damage mediated by Meth. Interestingly, treatment of cells with Meth resulted in increasing expression of Kv1.3 rather than Kv1.5, at both mRNA and protein level, which is partially blocked by MgTx. Furthermore, Meth also stimulated a significant increased expression of IL-6 and TNF-α at protein level, which was significantly inhibited by MgTx. Taken together, these results demonstrated that Kv1.3 was involved in Meth-mediated microglial damage, providing the potential target for the development of therapeutic strategies for Meth abuse.

## Introduction

Abuse of the illegal psychostimulant, methamphetamine (Meth), has become an international public health problem with an estimated 15–16 million users (United Nations office on Drugs and Crime, 2007). Meth abuse is associated with a number of negative consequences in humans, including renal and liver failure, cardiac arrhythmias, heart attacks, strokes, degenerative diseases and seizures [1], [2], [3]. Compelling evidence indicated that this psychostimulant causes damage in the brain, manifesting as degeneration of monoaminergic cells in the brains of Meth abusers. This damage may partly attributed to the depletions of dopamine and serotonin transporters, inhibition of tyrosine hydroxylase, reduction in function of the dopamine transporter, vesicle monoamine transporter and serotonin transporter markers [4], [5], [6], [7], [8].

Apart from the direct neural injuries, some indirect factors, such as microglial over activation and apoptosis, were also involved in neuronal damage [9]. Microglia, the resident immune cells within the central nervous system, were involved in regulatory processes critical for development, maintenance of the neural environment, response to injury, function in immune surveillance in the intact brain. Resting microglial cells secret neurotrophic factors to support neuronal function and survival [10]. However, over activation of microglia can lead to microglial apoptosis, contributing to the uncontrolled inflammatory response [11].

A positron emission tomography study has demonstrated prominent microglial over activation in the midbrain, stratum, thalamus and insular cortices in Meth abusers [12]. It was recently reported that Meth also exerts toxic effects on microglia; however, the mechanisms underlying this process are still unclear. Voltage-gated K^+^ channels have recently recognized as the promising targets for the therapy of neurological disorders [13]. Previous study has reported that several types of K^+^ channels were expressed in microglial cells, and the physiological properties as well as the expression level of these channels undergo dramatic changes attributed to microglial activation [14]. Of all, two candidates, namely Kv1.3 and Kv1.5, gained more attention, since Kv1.3 and Kv1.5 are expressed and comprised most of the Kv currents [15]. Pannasch et al found that Kv1.3 and Kv1.5 displayed distinct functions in microglia, the former is associated with microglial proliferation, while the latter involving non-proliferation[16]. Fordyce et al reported that activation of microglia induced by lipopolysaccharide (LPS), resulted in the increased expression of Kv1.3 rather than Kv1.5 at transcriptional level. [17]. Furthermore, blocking microglial Kv1.3 inhibited NADPH-mediated respiratory burst and ameliorated the neural death [18]. HIV-1 glycoprotein gp120 induced microglial activation with concomitant the increased expression of Kv1.3. Interestingly, treatment of Kv1.3 specific antagonist margatoxin, markedly mediated the neurotoxin production from microglia, such as CINC2, CINC3, IL-1α, IL-6 and TNF-α [19]. However, whether Meth exerts effects on the Kv1.3 expression and subsequently contributed to microglia damage remains largely unknown. Therefore, we will examine the effect of Meth on the microglia damage with emphasis on the role of potassium channel.

## Materials and Methods

### Animals

All experiments involving animals and tissue samples were conducted in accordance with the guide for the Care and Use of Laboratory Animals of the National Institutes of Health (NIH) (USA), and all procedures were approved by the Institutional Animal Care and Use Committee (IACUC) of Nanjing Medical University (China) (Permit Number: 20110521).Pregnant Sprague-Dawley rats were purchased from the Animal Center of Jiangsu Province, Nanjing, China (SCXK (Su) 2002-0031). All animals were housed in independent ventilation cages (IVC) at an ambient temperature of 23±1°C and humidity of 55±5% with 12 h light/dark cycle. They were allowed access to diet and tap water adlibitum.

### Materials

Methamphetamine was purchased from National Institute for Food and Drug Control (Beijing, China), aliquots of Meth were kept as 100 mM stock solution at −80°C. All chemicals, unless otherwise specified, were from Sigma.

### Primary microglial cell cultures

Pregnant Sprague-Dawley rats were anaesthetized with isoflurane and embryonic rats (18-day-old) were dissected out under sterile conditions. The cortical tissues were dissected in HBSS solution (Gibco, USA) and digested with 0.25% tripsin (Hyclone, USA). For microglia differentiation, isolated cells were planted into T75 flasks in high-glucose Dulbecco's modified Eagle's medium (Hyclone,USA) supplemented with 10% FBS, 2 mM L-glutamine, 1% penicillin/streptomycin, and 1 µg/ml MCSF(R&D,USA). After 10 days in culture, flanks were gently shaken to detach microglial cells then the cells were plated in a fresh T75. After 10 days in culture, OX-42 antibody (AbD Serotec, USA), a marker for microglia CR3/CD11b receptors was used to label the microglial cells while DAPI was used for nucleus staining. The purity of the microglia can be defined as follows: purity (%)  =  OX-42 staining cells/DAPI staining cells×100%.

### Immunocytochemistry

The microglial cells were fixed with methanol, after several washes in 0.1 M PBS, cells were permeabilized in PBS containing 0.3% Triton X-100 (ZSGB-BIO, Beijing) for 30 min, rinsed in PBS and then pre-incubated with 10% goat serum in PBS for 60 min at room temperature. The cells were then incubated in a solution of OX-42 antibody diluted in PBS overnight at 4°C. Then TRITC -conjugated goat anti-rabbit (1∶200 dilution; ZSGB-BIO, Beijing) were applied for 2 h at room temperature. Nuclei were stained with the fluorescent nucleic acid dye DAPI (1∶500 dilution; Vector, USA). After several washes, outgrowth cells were visualized using fluorescence microscope (Olympus IX70, Japan), adapted with a Mercury lamp (Olympus, Japan). The images were processed with the Image-pro Plus program.

### Whole cell patch clamp recording

Microglial cells were incubated with Meth for 24 h, then voltage-gated K^+^ currents were recorded with whole-cell patch clamp techniques by a patch-clamp amplifier (Axon200B, Molecular Devices, USA) the signals were filtered at 1 kHz and digitized at 5 kHz using a Digidata 1440A digitizer. The current and voltage traces were recorded using pCLAMP 10.2 data acquisition/analysis system.

The standard external solution contained (in mM): 150 NaCl, 4.5 KCl, 2 CaCl_2_, 1 MgCl_2_, 5 Hepes, 11 glucose, pH was adjusted to 7.4 by NaOH. The pipette solution contained (in mM):150 KCl, 2 MgCl_2_, 1 CaCl_2_, 11 EGTA, 10 Hepes, pH was adjusted to 7.3 by KOH. The resistance between the recording electrode filled with pipette solution and the reference electrode was 5–8 MΩ. Capacity transients were cancelled as much as possible, and voltage errors were minimized with series resistance compensation at 80–90%. Leakage current was digitally subtracted, while liquid junction potential was corrected throughout all experiments. Voltage-gated outward K^+^ currents were evoked by a series of +10 mV voltage steps to potentials between −70 and +30 mV from a holding potential of −70 mV.

### CCK-8 and TUNEL assay

Approximately 10^4^ microglial cells were plated per well in a 96-well plate and incubated with Meth for 24 h, then 10 µL Cell Counting Kit-8 Reagent (DojinDo, Japan) was added to each well. After incubation at 37°C for 4 h, absorbance was measured at a wavelength of 450 nm using a microplate reader (TECAN M200,USA). Microglial cells apoptosis was detected by the *in situ* cell death detection kit, AP (Roche Applied Science, USA) according to the manufacturer's instructions. In brief, 0.2×10^6^ cells were planted on coverslips in 12-well plates, then the cells were incubated with Meth for 24 h, after that, the cells were washed, and fixed with 4% paraformaldehyde, followed by permeabilizing with 0.2% Triton X-100 in ice for 20 min. Then the cells were processed with TUNEL staining (Green). ProLong Gold antifade reagent (Molecular Probes, USA) with 40, 60-diamidino-2-phenylindol (DAPI) was added to stain cell nuclei. Cells were visualized by fluorescence microscope (Olympus IX70, Japan), adapted with a Mercury lamp (Olympus, Japan).The apoptosis cells were counted and normalized to the total number of nuclei stained with DAPI.

### Quantitative real-time RT-PCR analysis

Total RNA was extracted from primary cultures using the Trizol Reagent (Invitrogen Life Technologies, USA) according to the manufacturer's instructions. 2 µg RNA reverse transcribed and was PCR-amplified using the Access RT-PCR system (Promega, USA) according to the manufacturer's instructions. Real-time PCR using Takara (Takara, Japan) was carried out on the 7300 System (ABI) for the detection of PCR products. The temperature profile was as follows: 94°C for 4 min, followed by 35 cycles of 94°C for 30 s, 59°C for 45 s, and 72°C for 45 s and a final extension phase of 10 min at 72°C.β-actin mRNA was used as the internal control for each sample since it was consistently expressed in all the cells. PCR primers were as follows: Kv1.3, forward primers: 5′- AGTATATGGTGATCGAAGAGG, reverse primers: AGTGAATATCTTCTTGATGTT; Kv1.5, forward primers: TCCGACGGCTGGACTCAATAA, reverse primers: CAGATGGCCT TCTAGGCTGTG; β-actin, forward primers: TCAAGAAGGTGGTGAAGCAG, reverse primers: AGGTGGAAGAATGGGAGTTG.

### Enzyme linked immunosorbent assay

Microglial cells were incubated with Meth for 24 h, concentrations of TNF-α, IL-6 (R&D systems, USA)and iNOs (Jiancheng Corporation, China) in the culture supernatants were measured by ELISA according to the manufacturer's instructions.

### Western Blot analysis

Cell lysates were prepared by using the RIPA (Radio-Immunoprecipitation Assay) buffer with with Protease Inhibitor Cocktail (Sigma, USA) and then incubated for 30 min at 4°C. The protein concentration was determined by Pierce BCA Protein Assay (Thermo Scientific,USA). Samples with the equal amounts of protein were loaded onto 10% sodium dodecyl sulfate–polyacrylamide gels, subjected to electrophoresis, and subsequently blotted onto 0.22 µM PVDF membrane (Millipore, USA). After blocking with 5% nonfat milk, the membranes were incubated with the polyclonal Kv1.3, Kv1.5 antibody (Alomone Labs,Israel). Mouse anti-β-actin (1∶5000, Sigma, USA) was used to detect reference protein expression. After incubated with HRP-conjugated anti-rabbit, or anti-mouse secondary antibody (1∶10000, Jackson ImmunoResearch Laboratories, USA) for 1 h at RT. Labeled proteins were visualized by Pierce ECL Western Blotting Substrate (Thermo Scientific, USA). Band densities were normalized to β-actin in each sample.

### Statistical analysis

All statistical analyses were performed with Statistical Package for the Social Sciences (SPSS) software (version 18.0,Armonk, NY, USA). Data are expressed as mean±SEM for all the experiments. The CCK-8, TUNEL, realtime-PCR and western-blot data were determined by one-way ANOVA and LSD multiple comparison procedure or the Student's t-test. All tests of statistical significance were two-sided and the statistical significance was set at P<0.05.

## Results

### Meth significantly enhanced the outward K^+^ currents

**Figure 1:**
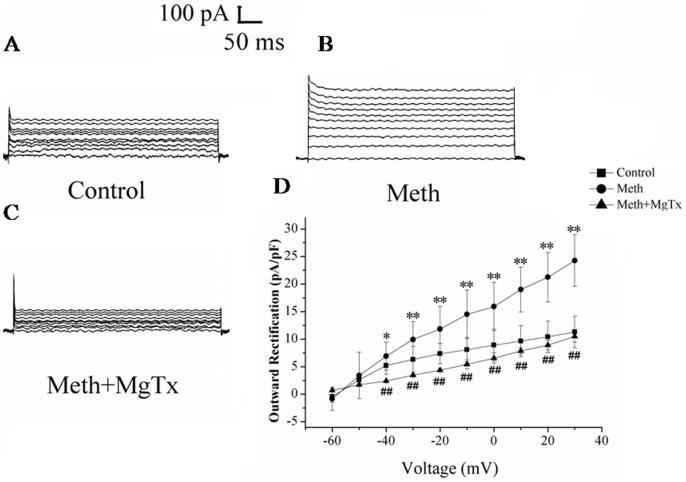
Meth significantly enhanced the outward K^+^ currents. The microglial cells were incubated with Meth (100 µM) for 24 h, then the K^+^ currents were recorded in (A) control group. (B) Meth treated group. (C) MgTx application before recording in Meth treated group. (D) The I–V plots of the K^+^ current density of microglial cells activated by voltage steps increments from a holding potential of −70 mV with intervals from −70 mV to +30 mV during 500 ms. *p<0.05 compared with control group (n = 10–15).

The microglial cells were incubated with 100 µM Meth for 24 h, then the outward K^+^ currents were recorded by the whole cell patch clamp recording. As depicted in Fig. 1, the K^+^ currents induced by Meth was obviously increased when compared with the currents recorded in the control group. However, the enhancement of the K^+^ currents mediated by Meth can be substantially retarded by MgTx, the Kv1.3 blocker.

### Kv blockers protected microglia against Meth-induced toxicity

**Figure 2:**
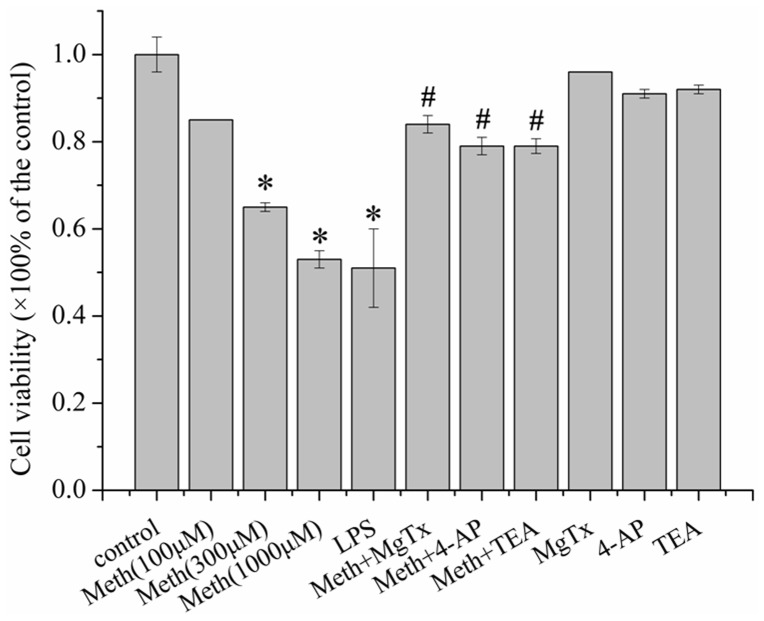
Kv1.3 channel involved in Meth mediated microglial damage. The cells were pre-incubated with 4-AP (1 mM), TEA (5 mM) and MgTx (10 nM) for 30 min before addition of Meth (300 µM). After the treatment of Meth for 24 h, the cell viability was evaluated by the CCK-8 assay. The toxic effect of Meth was dose-dependent, with a significant reduction at 300 and 1000 µM. The injury was partly retarded by the Kv antagonist 4-AP, TEA and MgTx. LPS (1000 ng/ml) was used for the positive control. Absorbance was measured at a wavelength of 450 nm using a microplate reader. Each data point represents mean ± S.E. of at least three separate experiments in which treatments were performed in quadruplicates. * indicates significant difference when the values were compared to that of the control. (*p<0.05) and ^#^ indicates significant difference when the values were compared to that in the Meth treated group (300 µM).

To assess the toxic effects of Meth on microglia, the CCK-8 and TUNEL assays were performed to evaluate the cell viability and apoptosis respectively. The cells were incubated with varied dose of Meth (0, 100, 300,1000 µM) for 24 h, then cell viability was evaluated by CCK-8 assay. As depicted in Fig. 2, Meth decreased the cell viability in a dose-dependent manner. We next explored whether its toxic effects could be related to Kv. Therefore, the broad spectrum Kv channel blocker, 4-AP, TEA and the Kv1.3 specific antagonist, MgTx were utilized. Our data suggested that all of the Kv blocker (4-AP, TEA and MgTx) protected against Meth induced microglial injury with 14.6%, 14.7% and 19.2% increments of cell viability respectively when compared with Meth treated group. In addition 4-AP, TEA and MgTx de novo did not affect cell viability.

**Figure 3:**
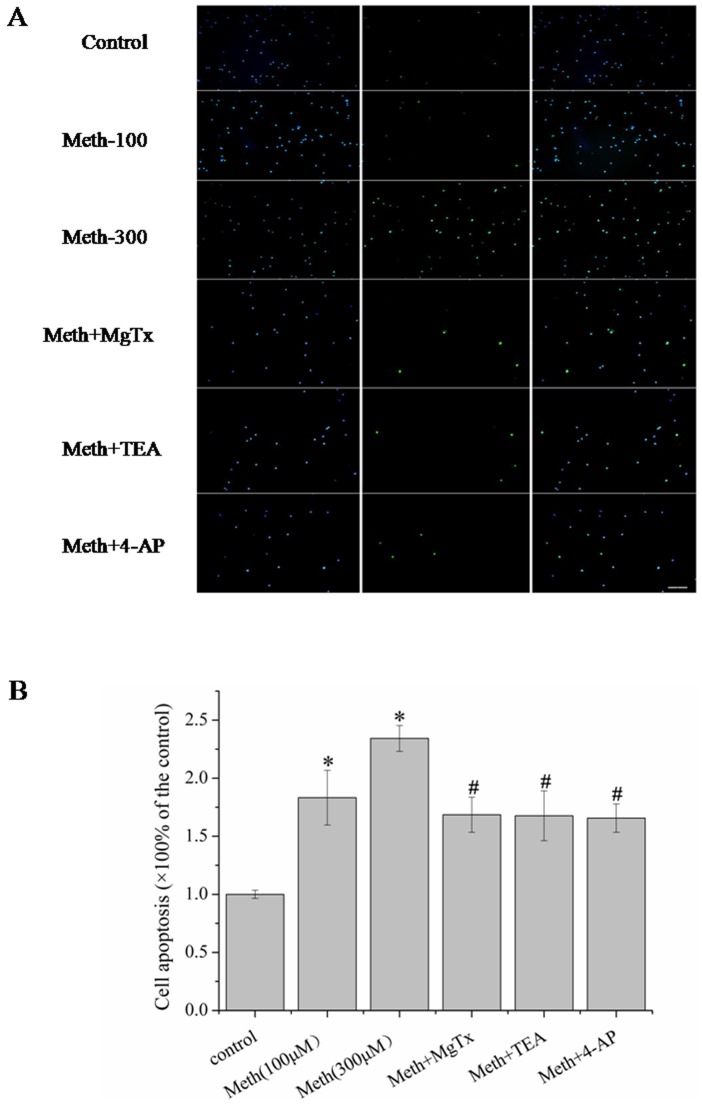
Kv1.3 was involved in Meth-induced microglial cell apoptosis, and the cell apoptosis was evaluated by performing the TUNEL assay. (A) Microglial cells were incubated with Meth (100,300 µM) for 24 h, and the apoptotic cells were markedly increased. (B) The cells were pre-incubated with 4-AP (1 mM), TEA (5 mM) and MgTx (10 nM) for 30 min before addition of Meth (300 µM). Apoptotic cells were substantially attenuated by the MgTx, or the broad spectrum Kv channel blockers 4-AP and TEA. The results are representative of three independent experiments performed in triplicate determinations. * indicates significant difference when the values were compared to that of the control. (*p<0.05) and ^#^ indicates significant difference when the values were compared to that of the Meth treated group (300 µM). Scale bars indicate 100 µm.

Having determined that Meth decreased cell viability of microglia, we next performed the TUNEL assay to further confirm the toxic effects of Meth on microglial cells. The cells were incubated with 0, 100 and 300 µM Meth for 24 h, then microglial cell apoptosis was subjected to the TUNEL assay. Our data revealed that Meth (300 µM) obviously increased the cell apoptosis (control: 1.00±0.04 vs Meth: 2.342±0.11), and the Kv blockers 4-AP (1.656±0.122), TEA (1.676±0.215) and MgTx (1.685±0.151) substantially attenuated Meth-induced cell injury (Fig. 3).

### Meth increased Kv1.3 mRNA expression in microglial cells

**Figure 4:**
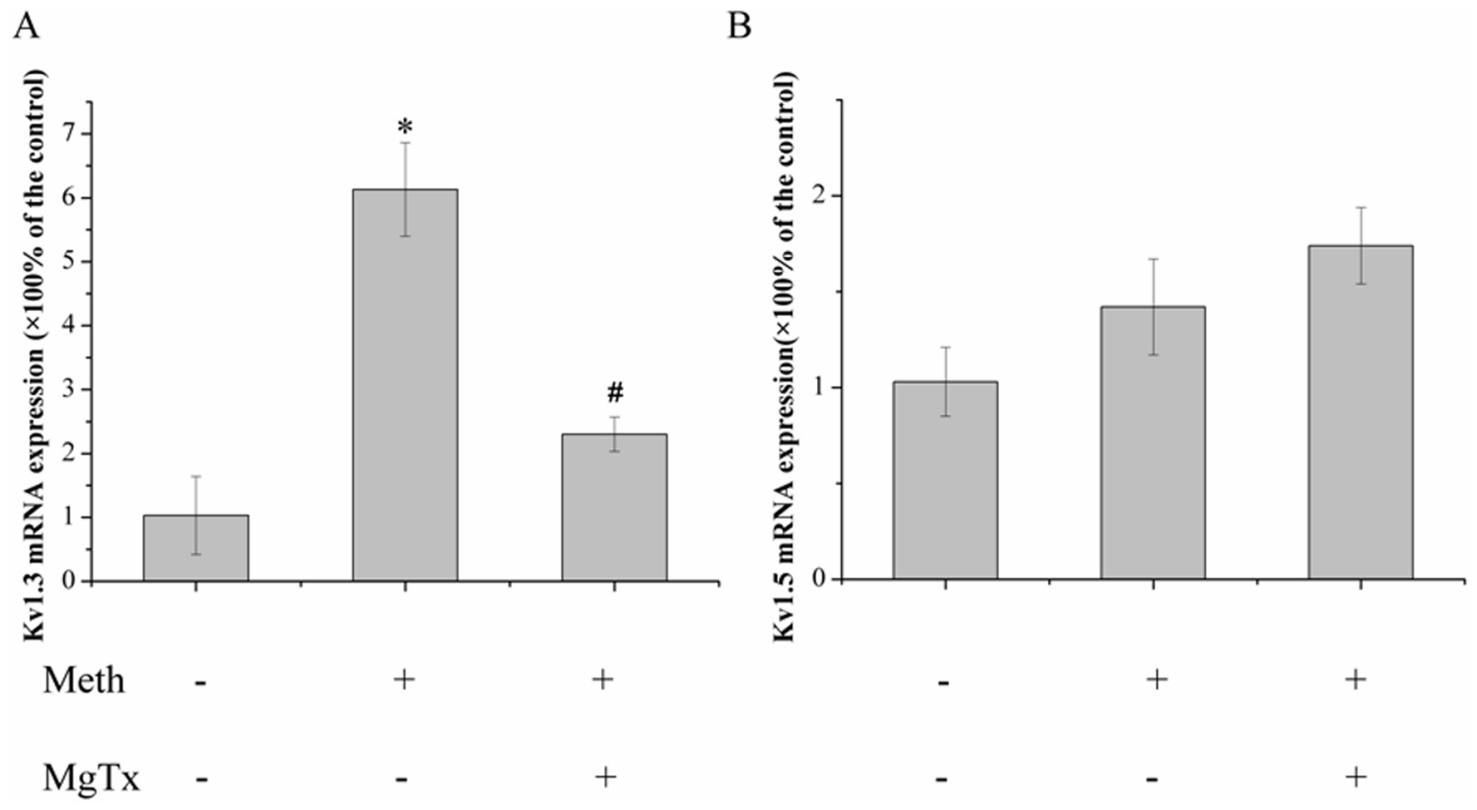
Meth increased Kv1.3 mRNA expression rather than Kv1.5. Microglial cells were incubated with Meth (300 µM) for 24 h, and the mRNA level was detected by realtime-PCR. (A) Meth significantly increased the Kv1.3 mRNA expression, while MgTx ameliorated the expression of Kv1.3 mediated by Meth. (B) Meth showed no obvious impact on Kv1.5 mRNA expression. Each data point represents mean±SD of mRNA levels from at least three separate experiments in which treatments were performed in triplicates.* and ^#^ indicate significant differences compared with the control and condition stimulated by Meth respectively (p<0.05 by ANOVA), each data represents mean±SD at least three separate experiments.

Since Meth-induced cell injury was ameliorated by MgTx, and recent evidence indicated that activated microglia express higher levels of Kv channels [19], then we next explored whether this effect could be related to Kv1.3 expression alteration. To assess the effects of Meth on Kv1.3 expression, Kv1.3 mRNA was monitored by realtime-PCR. β-actin was used for the housekeeping gene. As shown in Fig. 4A, the Kv1.3 mRNA expression increased by 6.1–fold in Meth treated group when compared with that in control group. However, the enhancement of Kv1.3 expression mediated by Meth was significantly retarded when the cells were treated with MgTx. Kv1.5, another major Kv channel in microglia, was also detected when treated with Meth by realtime-PCR, as shown in Fig. 4B, 300 µM Meth showed no obvious effect on Kv1.5 mRNA expression. Moreover, MgTx did not affect Kv1.5 mRNA expression mediated by Meth.

### Meth markedly up-regulated Kv1.3 protein expression other than Kv1.5

**Figure 5:**
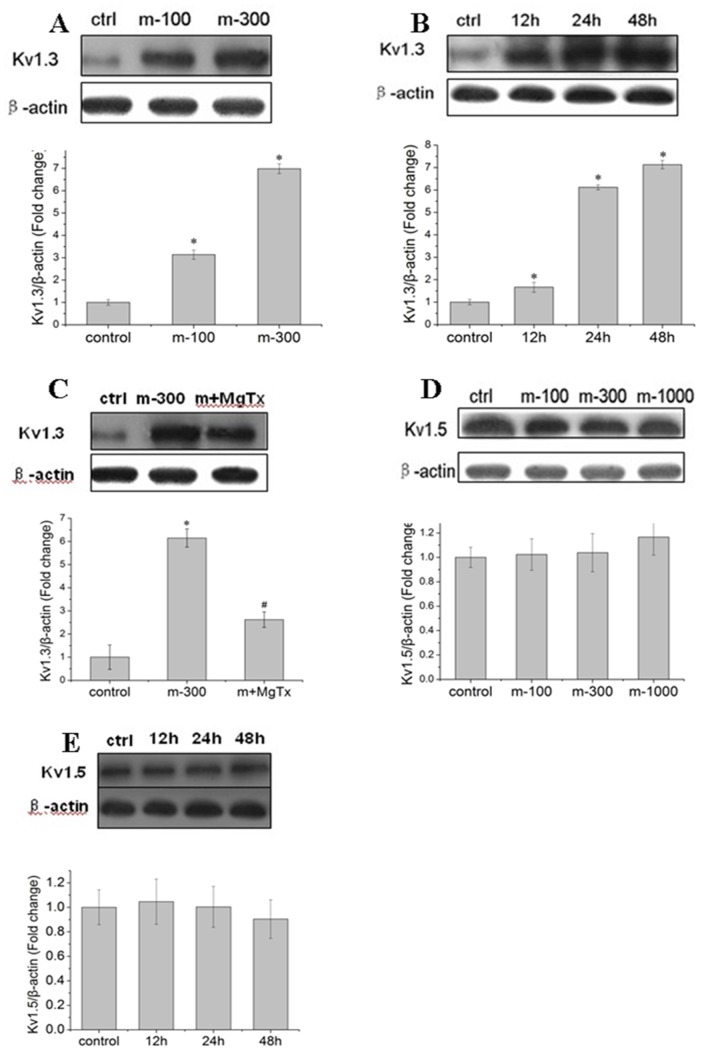
Meth increased Kv1.3 protein expression rather than Kv1.5. Microglial cells were incubated with Meth, and the level of Kv1.3, Kv1.5 proteins were detected by western-blot. (A) Meth (0, 100,300 µM) up-regulated Kv1.3 protein in a concentration dependent manner. (B) Meth (300 µM) obviously up-regulated Kv1.3 protein expression in 12, 24 and 48 h, and displayed a time-dependent way. (C) Meth mediated up-regulation of Kv1.3 protein level was partly attenuated by MgTx (10 nM). (D) and (E) Meth (300 µM) showed no impact on Kv1.5 protein level. * and ^#^ indicate significant differences compared with the control and condition stimulated by Meth respectively (p<0.05 by ANOVA), each data represents mean±SD at least three separate experiments.

As mRNA level is not always correspond with protein expression. So we next investigated the effects of Meth on the level of Kv1.3 and Kv1.5 protein expression. The microglial cells were incubated with Meth (0,100, 300 µM) for 24 h, and the level of protein expression was detected by western-blot. In agreement with the aforementioned findings, Meth (100, 300 µM) significantly up-regulated the Kv1.3, with 3.1-fold and 6.9-fold increment respectively (Fig. 5A). In addition, Kv1.3 expression in varied time point induced by Meth was also detected. As depicted in Fig. 5B, the increased Kv1.3 protein level was 1.7-fold, 6.0-fold and 7.1-fold respectively after incubated with Meth for 12, 24, 48 h when compared with the control. In line with the mRNA expression, MgTx substantially retarded Meth-mediated up-regulated Kv1.3 protein level (Fig. 5C). Likewise, Meth (0, 100, 300, 1000 µM) showed no impact on Kv1.5 protein expression (Fig. 5D and E).

### Kv1.3 was involved in Meth mediated inflammatory reaction

**Figure 6:**
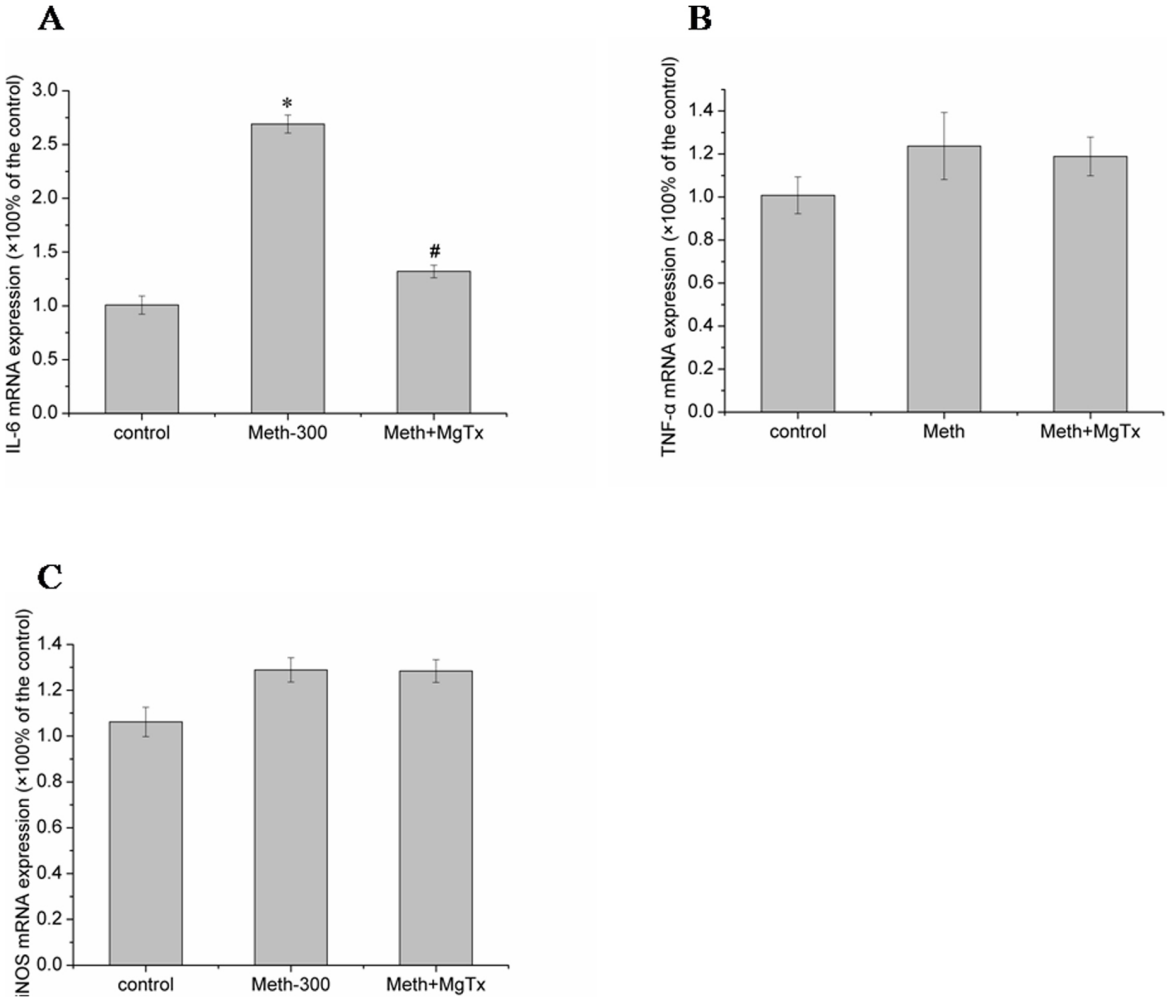
Effects of Meth on cytokines (IL-6, TNF-α and iNOS) mRNA expression. (A) Meth markedly increased IL-6 mRNA expression, while MgTx obviously inhibited IL-6 enhancement mediated by Meth. (B, C) Meth triggered a slight but not statistically significant increasing in TNF-α and iNOS mRNA level, and MgTx showed no obvious effects on TNF-α and iNOs mRNA when co-treated with Meth. Each data point represents mean±SD of mRNA levels from at least three separate experiments in which treatments were performed in triplicates.* and ^#^ indicate significant differences compared with the control and condition stimulated by Meth respectively (p<0.05 by ANOVA).

**Figure 7:**
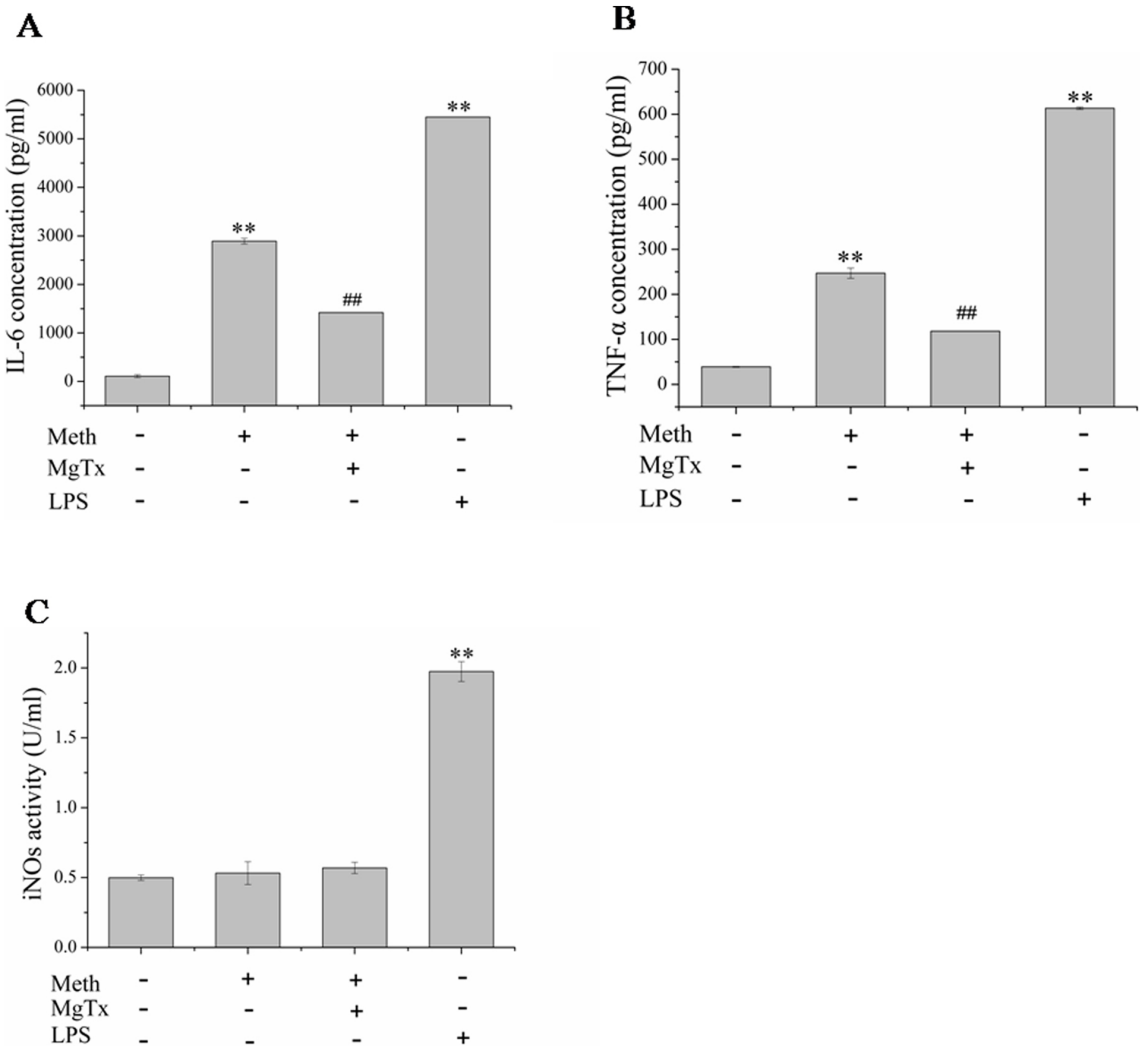
Effects of Meth on cytokines (IL-6, TNF-α and iNOS) secretion. (A, B) Meth markedly increased IL-6 and TNF-α secretion, while MgTx obviously inhibited IL-6 and TNF-α releasing mediated by Meth. (C) Meth exerted no obviously effect on iNOS activity. Each data point represents mean±SD of mRNA levels from at least three separate experiments in which treatments were performed in triplicates.* and ^#^ indicate significant differences compared with the control and condition stimulated by Meth respectively (p<0.05 by ANOVA).

Recent evidence implicating the importance of neuroinflammation mediated by Meth in damaged brain regions [20]. So we tested whether Kv1.3 was participated in Meth-mediated neuroinflammation. With the realtime-PCR and ELISA assay, we detected both of the mRNA and protein levels of several inflammatory factors induced by Meth. Our data showed that treatment of Meth contributed to up-regulation of IL-6 and TNF-α in both mRNA(Fig. 6) and protein(Fig. 7) levels. However,the up-regulation induced by Meth was obviously attenuated by co-incubated with MgTx (Fig. 6A, B and Fig. 7A, B). In addition, iNOs, another inflammatory factor, might be not affected by Meth, since both of the mRNA (Fig. 6C)and protein(Fig. 7C) levels were not markedly changed when treated with Meth.

## Discussion

It was well known that Meth activated the microglial cells. A positron emission tomography study using a radiotracer for activated microglia suggested that chronic self-administration of Meth resulted in reactive microgliosis in the brains of human Meth abusers [12]. The similar results were also indicated by Kitamura, with a significant increment of human glucose transportor5 (hGLUT), a marker for microglia [21].

The activated microglia may cause neurotoxicity through the excretion of varied cytokines and inflammatory factors that can initiated neuronal damage [10]. Our results indicated that Meth, with a concentration approximated to levels found in blood, urine or tissue samples of METH users [22], markedly increased the microglial cell damage, and facilitated the cytokines excretion. These data were in line with the report made by Tocharus that Meth-induced microglial cell line death and facilitated IL-6 mRNA expression [23]. However, the concentration of Meth which induced cell death and cytokines excretion they used, is much higher than ours, which may be attributed to the different cells.

Increasing evidences indicated a critical role for K^+^ channels in regulating cell death. Yu et al. suggested that the valinomycin, a K^+^ ionophore that allows K^+^ efflux based on the K^+^ electrochemical gradient, can induce apoptosis in neurons. By contrast, raising extracelluar K^+^, can inhibit neuron apoptosis induced by valinomycin [24]. Other Kv channel blockers, such as 4-AP and TEA have also been shown to suppress K^+^ channels and therefore prevent neuronal cell death and apoptosis [25]. For microglia, K^+^ channels were involved in several negative effects, such as cell activation, respiratory burst and inflammatory factors excretion [10], [18]. Our results, in the present study, indicating that Meth significantly increased the outward K^+^ currents, and resulted in efflux of the K^+^. However, several subtypes of the K^+^ channels might be involved in the outward currents mediated by Meth. Of all, two candidates, namely Kv1.3 and Kv1.5 were highly expressed and recognized as displaying distinct functions in microglial cells. Fordyce et al demonstrated that when the microglial cells were activated by LPS, the activated cells killed postnatal hippocampal neurons through a process that requires Kv1.3 channel activity, since down-regulated the expression of Kv1.3 or blocking Kv1.3 in microglia reversed the neural damage [17]. In other cells, like T cells, Kv1.3 activity has been identified as crucial during T cell activation and involved in inflammatory factors production as well [26]. For Kv1.5, few works to date has been reported on the issue of the relationship between Kv1.5 and cell damage. In addition, in rat microglia, Kv1.3 rather than Kv1.5 currents was detected by patch clamp [17]. Accordingly, in the present study, we focused on the effects of Kv1.3 in Meth-mediated microglial damage. Our results showed that pre-treated with MgTx, the Kv1.3 specific antagonist, substantially attenuated Meth- induced microglial damage. Moreover, the non-specific Kv blockers, 4-AP and TEA, obviously protected against Meth induced microglial damage. These data together, implying Kv1.3 might be a potential target for Meth.

Microglia expresses several types of Kv channels and the levels of their expression undergo dramatic changes during the process of activation. A study on the toxic effects of the HIV glycoprotein gp120 on microglia performed by Xiong et al indicated that gp120 markedly up-regulated Kv1.3 expression in both levels of mRNA and protein [19]. In accord with the aforementioned findings, our results revealed that Meth significantly enhanced Kv1.3 expression in a dose and time dependent manner, suggesting a specific effect of Meth on Kv1.3. In parallel, MgTx, the Kv1.3 blocker, obviously down-regulated Kv1.3 expression in both mRNA and protein levels which was agreed with the data that Kv1.3 inhibition protected against Meth-mediated cell damage. However, the mechanism for Kv1.3 expression alteration mediated by MgTx is unknown in the present study and deserved further research. Moreover, the Kv1.5 expression in both mRNA and protein were also detected, as depicted in Fig. 4 and 5, 300 µM Meth seems to exert no obvious effects on Kv1.5, since both of the Kv1.5 mRNA and protein levels were not markedly changed.

Microglial cells function as a source of neurotoxin in many infections, inflammatory brain disease. Ample evidence showed a neuropathological role of microglia had been postulated in most, if not all, neuroinflammatory or neurodegenerative diseases [27], [28], [29], [30]. The activated microglia produce a myriad of inflammatory mediators implicated in neuronal damage. So we detected the expression of the most common cytokines such as IL-6, iNOs and TNF-α. Our results indicated that Meth significantly enhanced the IL-6 and TNF-α expression in both mRNA and protein level, while iNOs expression was not obviously changed. Above data supported the work performed by Robson that Meth induced IL-6 mRNA increasing [20]. Studies have shown that Kv1.3 was implicated in cytokines production. One study with cytokine array blots assay showed that MgTx decreased the HIV coat protein gp120- induced cytokine production, such as IL-6 and TNF-α [19]. To explore whether Kv1.3 was involved in this process, we pre-treated the microglial cells with Kv1.3 specific blocker MgTx before incubating with Meth. Our results showed MgTx obviously ameliorated Meth-mediated IL-6 and TNF-α production. In addition, the level of iNOs mRNA was not obviously affected by MgTx when co-incubated with Meth.

In our study, we have focused exclusively on the role of Kv1.3 in Meth-induced microglial cell damage, and this process can be selectively blocked by the highly specific inhibitor MgTx. These finds are in agree with other reports that the Kv channel are the promising therapeutic targets not only for Meth abusing, but also for the treatment of other neurodegenerative disorders. However, we can not rule out the possibility that other K^+^ channel types are additionally involved in Meth-induced microglial cell damage and/or cytokines production, and furthermore, more work was required for elucidating the mechanisms of the effects of Meth on Kv1.3 and subsequently contributing to microglial cell death.
